# Protecting frontline healthcare workers should be the top priority in low-resource health systems: Bangladesh and COVID-19

**DOI:** 10.1017/ice.2020.208

**Published:** 2020-05-08

**Authors:** Md. Zakiul Hassan, Mohammad R. Monjur, Ashley Rene Styczynski, Mahmudur Rahman, Sayera Banu

**Affiliations:** 1 Programme for Emerging Infections, Infectious Diseases Division, icddr,b, Dhaka, Bangladesh; 2 University of Newcastle, New South Wales, Australia; 3 Division of Infectious Diseases and Geographic Medicine, School of Medicine, Stanford University, Palo Alto, California, United States of America

*To the Editor*—In April, the World Health Organization emphasized that the global shortage of personal protective equipment (PPE) is one of the most urgent threats sabotaging our ability to control the coronavirus 2019 (COVID-19) pandemic. As high-resource health systems around the world struggle to supply adequate PPE to their frontline healthcare workers (HCWs),^1^ it is likely that HCWs in low- and middle-income countries (LMICs) will bear the brunt of this global supply chain shortage. In Italy, 10% of the total confirmed COVID-19 cases have been among HCWs, and 105 HCWs have died.^2^ In the United States, state health departments similarly revealed a high proportion of cases among HCWs: Ohio (20%), Oklahoma (10.6%) and Pennsylvania (4.4%).^3^ Bangladesh reported its first HCW death on April 15, with 100 doctors and 57 nurses infected, and these numbers are likely to increase significantly as the country ramps up its testing efforts.^4^ Given the existing shortage of HCWs in Bangladesh, infection and subsequent absenteeism of HCWs from an already stretched workforce could leave Bangladesh grossly unprepared for the impending peak of the crisis.

Frontline HCWs in Bangladesh and other LMICs are particularly vulnerable to SARS-CoV-2 transmission because they work in overcrowded environments and have poor infection prevention and control (IPC) mechanisms—2 major risk factors for nosocomial infection transmission. In Bangladesh’s capital, Dhaka (one of the most densely populated cities in the world), a single-center study demonstrated a median of 4 people per 10 m^2^ of floor space.^5^ The close proximity between patients, caregivers, and HCWs can serve as a dangerous vehicle for rapid viral spread, placing HCWs in Bangladesh at an especially high risk of SARS-CoV-2 transmission. Further exacerbating the situation is poor infection prevention and control (IPC) practices at baseline; <2% of HCWs were compliant with recommended hand hygiene practices in a national survey, a result of both inadequate infrastructure and lack of IPC training.^6^ Many healthcare facilities in LMICs face a similar scenario; one study reported that 50% of healthcare facilities in LMICs lack piped water and 39% lack handwashing soap.^7^ Frontline HCWs should be equipped with the resources needed to create a safe environment for themselves and their patients, which includes access to hygiene measures and PPE in addition to adequate training on the application of both. Given the lack of PPE and the number of positive COVID-19 cases rising daily, HCWs are often forced to share PPE, to reuse disposable PPE without appropriate decontamination, and to rely on cloth masks that are inferior to medical masks. Moreover, the prospect of being unduly exposed to the virus has sparked widespread anxiety among medical professionals; many doctors have taken leave without notice and refuse to treat patients with confirmed or suspected COVID-19.

While awaiting essential PPE, environmental and administrative controls should be put in place to minimize HCW exposure. HCWs should strictly follow established protocols to assess, triage, and cohort patients. Additionally, trainings based on appropriate IPC practices, such as put forth by the Centers for Disease Control and Prevention (CDC), should be required for HCWs caring for patients with COVID-19. To reduce HCW shortages, staff inadvertently exposed to patients with COVID-19 could perform diligent self-monitoring and universal mask wearing, with isolation only if signs develop, as has been done in Singapore. Furthermore, rational use of PPE can be applied when the “gold standard” is not available. For example, the CDC recommends N95 respirator plus a face shield or a powered, air-purifying respirator (PAPR) when caring for patients with COVID-19. Given the extremely short supply of both in Bangladesh and other LMICs, other strategies should be employed, such as the use of medical masks on both patients and providers, which is supported by a recent randomized trial showing equally effective infection prevention.^8^

Despite these measures, a significant number of HCWs will almost invariably become infected, given the trend even in high-resource settings. Actively testing HCWs for SARS-CoV-2 will be key in swiftly identifying, isolating, supporting, and reintroducing infected HCWs following recovery. Given the short supply of testing kits, we believe that HCWs should be given priority for testing in LMICs. Testing symptomatic HCWs and those exposed to patients with COVID-19 will enable staff who have been unnecessarily quarantined to swiftly return to work if they test negative. One sample from the United Kingdom identified that only 1 in ~7 HCWs in self-isolation were actually positive for the virus.^9^

In addition to risking their physical well-being, frontline HCWs are enduring significant emotional burden both at work and home. A cross-sectional study across 34 hospitals in China found that frontline HCWs experienced symptoms of depression, anxiety, insomnia, and distress when directly engaged with diagnosing and managing patients with COVID-19.^10^ In part, they fear infecting their families, which is particularly problematic in Bangladesh where multifamily dwellings that preclude staying in a separate room are the norm. Psychosocial support must be offered to HCWs as they shoulder the weight of the epidemic, putting their lives at risk to help those who have been affected, and often encountering social stigma because they are presumed to be infectious. Fostering resilience among HCWs during such trying times and beyond can be achieved through routine peer support programs, preparation for potential moral dilemmas they may face, hotlines and crisis support, active monitoring of HCWs, and the availability of psychological therapy as needed.
